# Objectively measured physical activity during pregnancy: a study in obese and overweight women

**DOI:** 10.1186/1471-2393-10-76

**Published:** 2010-11-29

**Authors:** Catherine McParlin, Stephen C Robson, Peter WG Tennant, Hervé Besson, Judith Rankin, Ashley J Adamson, Mark S Pearce, Ruth Bell

**Affiliations:** 1 Newcastle-upon-Tyne Hospitals NHS Foundation Trust, Newcastle upon Tyne, UK; 2 Institute of Cellular Medicine, Newcastle University, Newcastle upon Tyne, UK; 3 Institute of Health and Society, Newcastle University, Newcastle upon Tyne, UK; 4 MRC Epidemiology Unit, Institute of Metabolic Science, Cambridge, UK

## Abstract

**Background:**

Obese and overweight women may benefit from increased physical activity (PA) during pregnancy. There is limited published data describing objectively measured PA in such women.

**Methods:**

A longitudinal observational study of PA intensity, type and duration using objective and subjective measurement methods. Fifty five pregnant women with booking body mass index (BMI) ≥ 25 kg/m^2 ^were recruited from a hospital ultrasound clinic in North East England. 26 (47%) were nulliparous and 22 (40%) were obese (BMI ≥ 30 kg/m^2^). PA was measured by accelerometry and self report questionnaire at 13 weeks, 26 weeks and/or 36 weeks gestation. Outcome measures were daily duration of light, moderate or vigorous activity assessed by accelerometry; calculated overall PA energy expenditure, (PAEE), and PAEE within four domains of activity based on self report.

**Results:**

At median 13 weeks gestation, women recorded a median 125 mins/day light activity and 35 mins/day moderate or vigorous activity (MVPA). 65% achieved the minimum recommended 30 mins/day MVPA. This proportion was maintained at 26 weeks (62%) and 36 weeks (71%). Women achieving more than 30 mins/day MVPA in the first trimester showed a significant reduction in duration of MVPA by the third trimester (11 mins/day, p = 0.003). Walking, swimming and floor exercises were the most commonly reported recreational activities but their contribution to estimated energy expenditure was small.

**Conclusion:**

Overweight and obese pregnant women can achieve and maintain recommended levels of PA throughout pregnancy. Interventions to promote PA should target changes in habitual activities at work and at home, and in particular walking.

## Background

The prevalence of obesity in the maternal population has increased sharply, reflecting the trend in the wider population [1,2]; one in six women now enter pregnancy already obese[3]. Maternal obesity is associated with increased risk of a range of adverse pregnancy outcomes[4-9]. Obese women are also more likely to retain weight gained during pregnancy[10]. There is increasing interest in the promotion of physical activity (PA) during pregnancy, not only in relation to maintaining energy balance and reducing excessive gestational weight gain, but also for its potential to improve pregnancy outcome for both mother and infant [11-18]. Professional bodies recommend that guidelines for the non-pregnant population of 30 minutes or more of at least moderate intensity activity daily remain appropriate throughout pregnancy. Moderate PA increases the heart and respiratory rate but the individual should still be able to hold a conversation. A common example is brisk walking [19-21].

Despite these recommendations there are limited data describing contemporary PA levels among pregnant women. Available data are mostly based on self-report, which may over-estimate activity [13,14,22-24], [25]. Much activity in pregnancy is low intensity and dominated by childcare and domestic tasks, activities which may be less well captured by questionnaires[23,25,26]. In recent years, objective methods for measurement of physical activity such as accelerometry, which have a high degree of validity for quantifying activity intensity and duration[27,28], have become available and are widely used [22,23,29], although few studies have used these methods to measure activity during pregnancy. Chasan-Taber et al used accelerometry to validate the Pregnancy Physical Activity Questionnaire in 54 women at various stages of pregnancy[22], while Rousham et al [29] measured activity in 58 women throughout their first pregnancy.

Information about habitual activity levels in obese and overweight pregnant women is lacking, although these women have the potential to benefit most from interventions to promote PA due to their increased risk of adverse outcome. This study assessed and compared the duration and intensity of objectively measured PA in overweight and obese pregnant women longitudinally in early and late pregnancy, and explored types of activity via self report questionnaire.

## Method

### Study population

Recruitment took place between 03 October 2007 and 31 January 2008. A favourable ethical opinion was obtained from Durham and Tees Valley 2 Research Ethics Committee (ref No.07/H0908/53). All pregnant women attending the Royal Victoria Infirmary, Newcastle Upon Tyne for a routine first trimester ultrasound scan were sent a participant information sheet with their appointment. Women with a booking body mass index (BMI) of 25 kg/m^2 ^or greater, and with a normal scan result, were approached for consent to participate. BMI was based on weight measured at booking by the community midwife or by the research midwife at recruitment. Women were excluded if they were less than 16 years old, attended for first scan after 14 weeks of pregnancy, had a multiple pregnancy or were unable to give informed consent in English.

**Figure 1:**
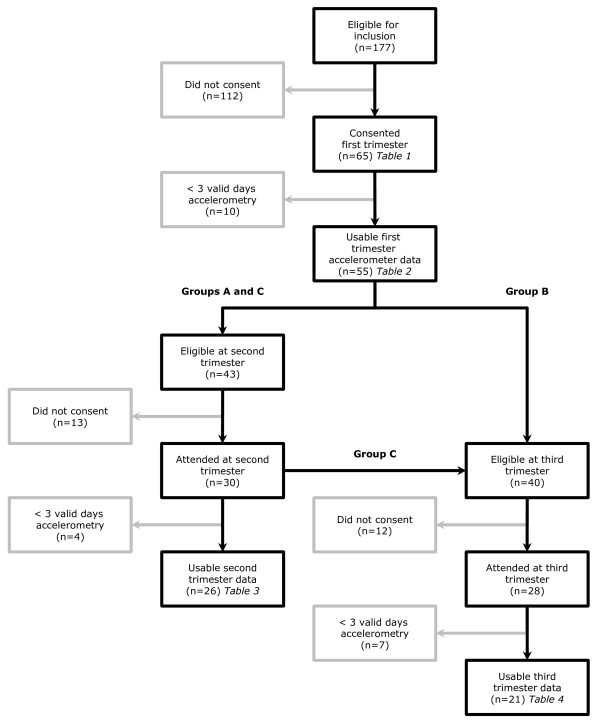
**Flow chart of participants' progress through the study**.

Participants were randomly allocated using an Excel random number generating programme into one of three groups. All women were invited to participate in the first data collection point immediately following recruitment. Invitation to subsequent data collection was on the basis of group allocation: group A were invited to participate at 26 weeks only, group B at 36 weeks only, and group C at both 26 and 36 weeks. A flow chart of women's progress through the study is detailed in Figure 1. Thus all women were invited to either two or three data collection points. This design was chosen to minimise attrition and maximise the numbers participating in later pregnancy.

Prior to the second and third measurements, hospital records were checked and women excluded from further participation if medical or obstetric complications had arisen.

### Data collection

Participants were asked to wear a GT1 M Actigraph accelerometer, http://www.theactigraph.com, for seven days. The accelerometer was attached to an elastic belt worn around the waist with the monitor positioned over the right hip. Women were instructed to start wearing the monitor as soon as practicable in the morning and to wear it for as much of the day as possible, removing it for washing and bathing, swimming and for bed at night. At the end of the seven day period the women were asked to complete a self report physical activity questionnaire.

### Accelerometry data processing

The accelerometer data files were processed using the MAHUFFE Software package http://www.mrc-epid.cam.ac.uk/Research/PA/Downloads.html. Sedentary behaviour was defined as less than 100 counts per minute, light activity as 100-1951 counts per minute, moderate intensity activity as 1952-5725 counts per minute and vigorous activity as more than 5725 counts per minute[27]. As the time spent in vigorous activity was very low, minutes of moderate and vigorous physical activity (MVPA) were combined to give one summary variable. An epoch length of 5 seconds was used. Runs of zero counts lasting more than 60 minutes were excluded, as it was considered the monitor must have been removed for this time. A valid day of recording was defined as one in which more than 500 minutes of monitored on-time were recorded in a 24 hour period; only women recording at least three valid days of accelerometry were included and only valid days were analysed.

### Questionnaire data

We used the Recent Physical Activity Questionnaire[30] (RPAQ). The RPAQ contains closed questions about activity in four main domains: at home, during work, for transport and during leisure time. For the purposes of this study, the time frame of reference for the RPAQ was modified from four weeks to one week, because it was anticipated that habitual levels of activity might change over short time frames during pregnancy.

Estimates of PAEE for the four domains were calculated by multiplying participation (hrs/day) by the metabolic cost of each activity in metabolic equivalents (METs) obtained from the Physical Activity Compendium. Total PAEE was calculated by summing PAEE in each domain[31]. The RPAQ also provided information regarding types of recreational activity. Questionnaires were double data entered by an independent data entry company and all analysis used the statistical package, SPSS Version 15 (SPSS Inc, Chicago IL).

### Statistical analysis

As PA variables were skewed, non-parametric statistics were used. Summary statistics are presented as the median and inter-quartile range (IQR). Differences between subgroups (e.g. nulliparous vs multiparous) were examined using the Mann-Whitney U test. Changes in activity between first and second trimester, and between first and third trimester, were assessed using the Wilcoxon signed ranks test. This was a paired analysis and therefore only women who completed both respective time points were included. P ≤ 0.05 was considered statistically significant.

### Ethical Approval

Ethical approval was granted by Durham and Tees Valley 2 Research Ethics Committee, 20^th ^August 2007. REC reference number: 07/H0908/53

## Results

### Participants

Sixty five of 177 (37%) eligible women consented to participate in the study. Fifty five (85%) completed three or more valid days of accelerometry measurement (median seven days) at the first data collection point (median 13 weeks of pregnancy, range 11-15). Of these 55 women, 26 (47%) were nulliparous and 22 (40%) were obese at booking (Table 1).

**Table 1:** Study participants

	First trimester *(n = 55)*	Second trimester *(n = 26)*	Third trimester *(n = 21)*
**Age (yrs)**			
Mean (SD)	30.7 (5.3)	31.3 (6.5)	32.8 (3.9)
**Gestation**			
Median (Range)	13 (11-15)	26 (25-28)	36 (34-37)
**BMI category**			
25-29.9 kg/m^2^	33 (60%)	13 (50%)	13 (62%)
≥ 30 kg/m^2^	22 (40%)	13 (50%)	8 (38%)
**Parity**			
Nulliparous	26 (47%)	12 (46%)	8 (38%)
Multiparous	29 (53%)	14 (54%)	13 (62%)
**Ethnic origin**			
White British	51 (93%)	24 (92%)	20 (95%)
Other/unknown	4 (7%)	2 (8%)	1 (5%)
**Employed in past week**			
Full time > 35 hours	20 (36%)	5 (19%)	3 (15%)
Part time up to 35 hrs	22 (40%)	14 (54%)	3 (14%)
Not working in previous week	13 (24%)	7 (27%)	15 (71%)
**Living with partner**			
Yes	49 (89%)	23 (88%)	21 (100%)
No	4 (7%)	3 (12%)	-
Missing	2 (4%)	-	-
**Education level:**			
GCSE or lower (16 yrs)	11 (20%)	4 (15%)	3 (14%)
A level/equivalent (18 yrs)	20 (36%)	12 (46%)	6 (29%)
Degree/post graduate	18 (33%)	7 (27%)	11 (52%)
Missing	6 (11%)	3 (12%)	1 (5%)

At the second data collection point (median 26 weeks, range 25-28), 43 women were eligible and 30 (70%) agreed to participate, of whom 26 (61%) recorded three or more valid days of accelerometry data (median six days). Forty women were eligible for the third data collection point (median 36 weeks, range 34-37). Two women had already delivered and one was excluded due to severe symphysis pubis pain. Twenty eight (68%) women agreed to participate and 21 (53%) recorded three or more valid days of accelerometry data (median six days). Ten of these women had also participated in the second data collection (Figure 1). All accelerometers were returned in working order.

Characteristics of women who participated in the second data collection were similar to those in the first trimester. Participants in the third trimester data collection were less likely to be working outside the home (29%) than participants in the first (76%) and second (73%) trimester (p < 0.001). Participants in the third data collection were more likely to be multiparous than first trimester participants (62% compared to 53%), and to have a degree or post graduate qualification (52% compared to 33%), although neither of these differences reached statistical significance.

### Physical activity in early pregnancy

The median duration of valid accelerometry recording at the first data collection point was 798 minutes per day (table 2). The median duration of non-sedentary time (i.e. time spent in activity of at least light intensity) was 165 minutes per day, and the median duration of MVPA was 35 minutes per day. Thirty-five women (63%) achieved more than the recommended 30 minutes per day of MVPA.

**Table 2:** Objectively measured and self reported physical activity in first trimester (median; interquartile range)

	All women *n = 55*	Nulliparous *n = 26*	Multiparous *n = 29*	*p value*
**Accelerometry data:**				
Total recorded time (mins/day)	798 (742 -835)	789 (736-782)	808 (743-836)	0.711
Sedentary time (mins/day)	631 (574-673)	631 (580-683)	620 (553-667)	0.613
Light activity (mins/day)	125 (97- 153)	106 (96-136)	135 (108-163)	0.032
MVPA (mins/day)	35 (28-51)	36 (27-50)	32 (27-52)	0.649
Counts per minute	261 (209-318)	266 (207-299)	248 (211-342)	0.933
Recording 30+ mins/day MVPA (number)	35 (63.3%)	19 (67.9%)	16 (47.1%)	0.168
**RPAQ data:**	*n = 57*	*n = 26*	*n = 31*	
Total (METhrs/day)	26.5 (24.9-29.2)	25.6 (24.3-27.2)	28.0 (25.8-30.0)	0.022
Home (domestic + childcare) (METhrs/day)	17.0 (13.7-20.4)	14.1 (12.6-16.0)	20.2 (17.0-21.5)	< 0.001
Transport (METhrs/day)	0.19 (0.09-0.63)	0.25 (0.12-0.84)	0.18 (0.04-0.57)	0.219
Work (METhrs/day)	9.4 (5.5-11.4)	10.6 (8.3-12.0)	7.5 (4.0-10.6)	0.007
Recreational activities (METhrs/day)	0.93 (0.12-1.9)	0.83 (0.5-1.6)	1.2 (0-2.5)	0.734

Multiparous women spent more time in light activity than nulliparous women (table 2; median 135 min/day compared to 106 min/day; p = 0.032). Otherwise there were no statistically significant differences in duration of sedentary time, MVPA, or in median counts/min, between nulliparous and multiparous women. There were no significant differences in duration and intensity of activity between overweight and obese women (data not shown).

Data from RPAQ indicated that 62% of self reported PAEE was related to activity at home and 34% at work. A very small proportion of self reported PAEE was attributable to recreational activity or to transport. The most commonly reported recreational activities were walking (29 women, 51%), swimming (11 women, 19%) and floor exercises (10 women, 18%). One woman reported cycling. Women in their second or subsequent pregnancy reported higher levels of activity within the home domain, which includes childcare, and lower levels at work compared with those in their first pregnancy. This reflects the higher proportion of multiparous women who were working part-time (18 (78%) compared to 6 (29%); p < 0.001).

### Changes in physical activity during pregnancy

In women with repeated measures, there were small but statistically significant decreases in total recorded time and sedentary time, at both the second and third data collection points (table 3 and Table 4).

**Table 3:** Change in physical activity levels between first and second trimesters (median, interquartile range)

	First trimester	Second trimester	Change first-second trimester	p value
**Accelerometry data:**		n = 26*		

Total recorded time (mins/day)	780 (725, 838)	742 (693, 800)	-44 (-91.4, 9.3)	0.018

Sedentary time (mins/day)	631 (570, 673)	585 (524, 637)	-25 (-84, 12.4)	0.023

Light activity (mins/day)	122 (95, 152)	122 (101, 154)	-9 min (-17.2, 12.0)	0.304

MVPA (mins/day)	36 (26, 66)	33 (27, 52)	-2 min (-8.9,+1.8)	0.269

Counts per minute	262 (199, 384)	259 (226, 338)	3 (-40.6, 34.8)	0.909

30+ mins/day MVPA (number)(%)	17 (65%)	16 (62%)	-	

**RPAQ^‡ ^data:**		n = 26*		

Total (METhrs/day)	26.4 (23.0, 29.0)	25.7 (22.8, 29.1)	-0.35 (-2.12, 1.28)	0.638

Home (METhrs/day)	16.4 (13.1, 20.5)	17.2 (13.0, 19.7)	0.29 (-1.41, 1.77)	0.990

Transport (METhrs/day)	0.25 (0.11, 0.66)	0.21 (0.07, 0.76)	0 (-0.07, 0.10)	0.906

Work (METhrs/day)	8.3 (5.7, 11.3)	8.0 (5.6, 10.1)	0 (-2.06, 0.04)	0.037

Recreational (METhrs/day)	0.71 (0.0, 1.25)	0.88 (0.28, 2.21)	0.36 (-0.37, 1.58)	0.055

*Paired analysis of those participating in first and second trimester data collection

^‡ ^Recent Physical Activity Questionnaire[30]

**Table 4:** Change in physical activity levels between first and third trimesters (median, interquartile range)

	First trimester	Third trimester	Change first-third trimester	p value
**Accelerometry data:**		***n = 21^†^***		

Total recorded time (mins/day)	817 (787, 866)	778 (727, 840)	-32.6 mins (-78.4, 9.2)	0.019

Sedentary time (mins/day)	670 (615, 701)	625 (547, 683)	-23.6 mins (-58.9, 7.0)	0.017

Light activity (mins/day)	129 (102, 155)	120 (105, 164)	3.8 (-21.9, 17.6)	0.986

MVPA (mins/day)	33 (29, 55)	33 (28, 35)	-8.4 mins (-21.7, 1.3)	0.050

Counts per minute	262 (213, 323)	247 (210, 287)	-1.5 (-89.7, 26.5)	0.274

30+ mins/day MVPA (number)(%)	15 (71%)	15 (71%)	-	

**RPAQ^‡ ^data:**		***n = 27^†^***		

Total (METhrs/day)	26.0 (24.6, 28.0)	23.5 (22.5, 27.6)	-1.85 (-4.40, 0.24)	0.027

Home (METhrs/day)	15.4 (13.3, 18.9)	20.5 (17.3, 21.9)	3.03 (0.72, 6.58)	< 0.001

Transport (METhrs/day)	0.5 (0.2, 0.9)	0 (0.0, 0.1)	-0.32 (-0.93, -0.06)	< 0.001

Work (METhrs/day)	9.6 (5.7, 10.7)	0 (0.0, 6.9)	-5.43 (-10.0, 0)	< 0.001

Recreational (METhrs/day)	0.9 (0.4, 1.6)	1.1 (0.0, 3.0)	0.20 (-0.43, 1.29)	0.174

^†^Paired analysis of those participating in first and third trimester data collection

^‡ ^Recent Physical Activity Questionnaire[30]

**Figure 2:**
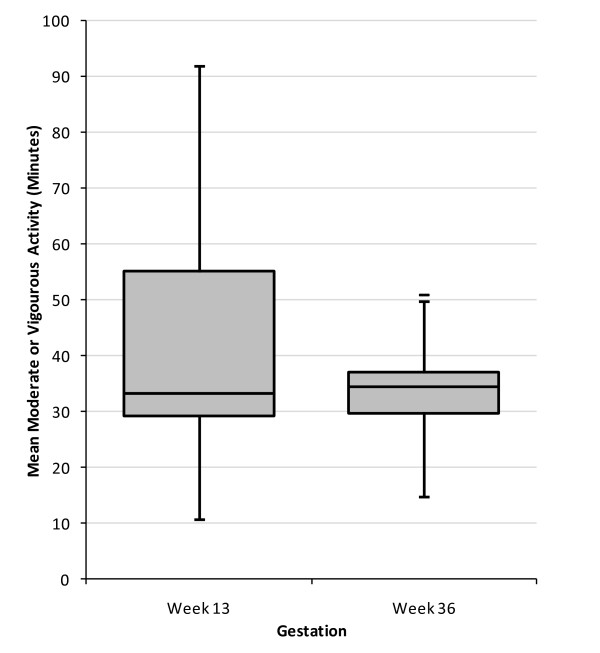
**Change in objectively measured moderate or vigorous activity between week 13 and week 36 of pregnancy**.^§^

In women with data for both first and third trimesters, there was no significant change in duration of light activity or in counts per minute. The overall median duration of MVPA was unchanged between the first and third trimester (33 minutes) and the proportion of women recording at least 30 minutes MVPA was also unchanged at 71%. There was however a reduction (median 8 minutes) in duration of MVPA (p = 0.05) (Figure 2) which was mainly confined to women who recorded longer durations of MVPA in the first trimester. In women who achieved at least 30 minutes of MVPA in the first trimester, the median reduction was 11 minutes (p = 0.003). These women also recorded a significant reduction in counts/min (change -49 counts p = 0.023).

Self reported data from RPAQ found no significant changes between 13 and 26 weeks in overall calculated PAEE. Between 13 and 36 weeks, overall self-reported estimated PAEE decreased by a median of 1.85 METhours/day (p = 0.027). Estimated PAEE at home increased whilst PAEE at work reduced. The proportion of women reporting participation in recreational walking and floor based exercise was largely maintained at all data collection points. Between 13 and 26 weeks of pregnancy the proportion reporting swimming increased (4 (15%) vs 7 (27%); p < 0.001).

## Discussion

This study describes the amount, intensity and type of habitual PA in overweight and obese pregnant women of mixed parity using both objective and self report measurement methods. To our knowledge, this is the first study to report objectively measured activity levels longitudinally during pregnancy in obese and overweight women. We found that accelerometry was well tolerated even in late pregnancy, and that PA levels did not differ between overweight and obese women. Women continued to meet recommended PA levels throughout pregnancy.

Over 60% of our overweight and obese subjects achieved more than the recommended 30 minutes per day of moderate or vigorous activity in early pregnancy. In a study of non-pregnant women (mean age 40.7 years, SD 6.4) assessed using the same methodology 34% achieved at least 30 minutes MVPA/day [32]. The 2008 Health Survey for England reported a mean of 31 minutes/day MVPA in overweight women aged 16-34 years, and 27 minutes/day in obese women [33]. Comparison with other studies in pregnancy is difficult due to differences in recording methods and definitions. A large telephone survey of pregnant women in the USA found that only 16% of pregnant women achieved recommended activity levels of at least 30 minutes of moderate activity on 5 days per week[34].

Thus, the proportion of women in our study achieving 30 minutes of daily MVPA was higher than might be anticipated. It should be noted that we did not analyse bouts of continuous MVPA, but rather included all accumulated episodes of MVPA occurring throughout the day. The proportion of women undertaking continuous five or ten minute bouts of MVPA is likely to have been substantially lower[32]. It is possible that our participants consciously or sub-consciously increased their PA whilst activity measurement was taking place; however, most women returned at least six valid days of recording, which compares favourably with many accelerometer studies. Only 37% of eligible women agreed to participate and these may have been biased to more active women; however the mean age, BMI and ethnic background of participants were comparable to women who declined. A third of participants were educated to at least degree level, and women of more advantaged socio-economic status are more likely to meet recommended PA levels than women in less advantaged socio-economic groups[33]. Moreover, those who declined participation may have been more likely to be either habitually inactive or affected by physical limitations related to pregnancy, and thus not inclined to enter a study with a focus on measuring PA.

Our sample size was relatively small and thus we had limited power to detect differences between subgroups such as obese and overweight women, and multiparous and nulliparous women. Non-significant results should therefore not be considered as proof of no effect. We restricted our study to women with uncomplicated pregnancies and are unable to provide information on PA levels in women with pregnancy complications. Three women were excluded from the third trimester measurement due to complications arising during pregnancy.

In this sample of overweight and obese pregnant women the duration of objectively measured light and MVPA, total recorded body movements (accelerometer counts/min), and the proportion achieving the recommended level of 30 minutes/day MVPA were relatively stable throughout pregnancy. However, women who recorded more than 30 minutes of MVPA in early pregnancy did reduce their duration of MVPA, by a median of 11 minutes per day, by the third trimester. Counts per minute also significantly reduced in this group. We are aware of only one other longitudinal study of objectively measured activity in pregnancy. Rousham et al reported that average counts per minute decreased substantially as pregnancy progressed[29]. In that study all participants were low risk nulliparous women, who may have had higher baseline activity levels than our participants.

In contrast to objectively recorded activity levels, there was a decline in overall self reported PAEE between the first and third trimesters. This is consistent with other studies using self reported measures of activity [26,35,36]. It should be noted that the RPAQ was not designed to measure changes over time and therefore these findings should be interpreted with caution[30]. Further, in the validation of a similar questionnaire[37] the home domain was less strongly correlated with objective measurement than other domains. In our study, this domain accounted for the majority of self-reported PAEE, and this may explain the discrepancy between changes in objectively reported activity and self reported activity. PAEE was calculated on the basis of published MET values from the general population; pregnancy-specific MET values are unavailable and we were unable to assess the extent to which this may have biased PAEE estimates.

As in other studies[34,38] the main recreational activities reported by the women during pregnancy were walking and swimming. These were also activities that were maintained or reported by more women in later pregnancy. Other authors have also reported that walking is maintained as pregnancy progresses[39,40].

We found that estimated self reported PAEE attributable to recreational activities was low compared to that attributed to activity at home and at work. Thus, maintenance of PA levels during pregnancy is likely to be determined mainly by habitual activities associated with daily domestic and work routines, rather than by participation in structured leisure time activities.

Between the first and third trimester there was an increase in self-reported PAEE attributed to home activities (including childcare) and a reduction in PAEE derived from work. This trend has also been reported by Derbyshire et al[41], who reported a higher proportion of self reported expenditure in the home in the third trimester. In contrast, Clarke[42] found that self reported estimated energy expenditure related to domestic activities remained static throughout pregnancy, however the participants were all women in their first pregnancy. The findings of the current study are consistent with substitution of home activity for work activity towards the end of pregnancy; the objective measurements suggest that overall light activity was maintained whilst higher intensity activities were reduced.

Multiparous women recorded a higher duration of light activity in early pregnancy, and self reported data suggested that this was attributable to activity in the home. Larger studies are needed to further explore differences between nulliparous and multiparous women and whether these differences are maintained as pregnancy progresses.

Our study demonstrates that measurement of activity by accelerometry is feasible and acceptable throughout pregnancy. A major benefit of objective measurement methods is that they are unlikely to produce biased measures, unlike subjective measures which quantify the individual's perception of activity, and therefore frequently over-estimate activity levels[43]. We selected a questionnaire which captured activity related to domestic and childcare tasks separately. Whilst this is an important contributor to habitual non-sedentary activity in pregnant women, it may be particularly subject to over reporting as women often perform more than one task at once, for example housework and childcare[44]. Accelerometry does not have the same limitations but the quality of the data is affected by compliance and acceptability of the device. Furthermore, accelerometry cannot capture activity related to swimming, which was reported throughout pregnancy, thus slightly under-estimating activity levels in those women who swam. Similarly, accelerometry is a poor method for capturing activity during cycling; however only one woman in our sample reported this.

At later time points, the accelerometers were worn for shorter periods of time and recorded sedentary time was reduced, suggesting that the monitor was worn less often during resting or sitting in the second and third trimesters. Nevertheless, the duration and number of days recording achieved in this study compare well with that reported in other populations [22,29]. There is no consensus about the most appropriate activity count cut off points for sedentary, light, moderate and vigorous PA levels in pregnancy. We chose those proposed by Freedson et al, which have been widely used in the non-pregnant adult population[27].

## Conclusion

Our study shows that it is possible for overweight and obese women to achieve the recommended 30 minutes of moderate activity throughout pregnancy, and we suggest this is a realistic aim for this group. Women who were more active in early pregnancy significantly reduced their recorded MVPA in late pregnancy. The reasons why some women reduce their activity levels, and methods to encourage maintenance of PA throughout pregnancy, require further investigation. Recreational activities appear to contribute little to overall habitual activity levels in this group of women, and therefore future studies should use measurement methods which capture overall habitual PA. Interventions to promote PA in pregnancy should target and support changes in habitual activities at work and home, and in particular walking.

## Competing interests

The authors declare that they have no competing interests.

## Authors' contributions

**CMcP **contributed to the study design, recruited participants and collected the data, analysed the data and wrote the first draft of the manuscript. **SCR **contributed to the conception and design of the study and interpretation of the results. **PT **provided statistical advice and contributed to the interpretation of the results. **HB **provided PAEE calculations from the raw RPAQ data. **JR **contributed to the design of the study and interpretation of the results. **MP **contributed to the design of the study, gave advice regarding statistical analysis and interpreted the results. **AJA **contributed to the design of the study and interpretation of the results. **RB **contributed to the conception and design of the study, analysis and interpretation of the data and writing of the manuscript. All authors read and approved the final manuscript.

## Pre-publication history

The pre-publication history for this paper can be accessed here:

http://www.biomedcentral.com/1471-2393/10/76/prepub
